# Age affects procedural paired-associates learning in the grey mouse lemur (*Microcebus murinus*)

**DOI:** 10.1038/s41598-021-80960-y

**Published:** 2021-01-13

**Authors:** Daniel Schmidtke

**Affiliations:** Institute of Zoology, University of Veterinary Medicine Hannover, Hannover, Germany

**Keywords:** Cortex, Hippocampus, Animal behaviour, Experimental models of disease, Translational research

## Abstract

The ability to associate memorized objects with their location in space gradually declines during normal aging and can drastically be affected by neurodegenerative diseases. This study investigates object-location paired-associates learning (PAL) in the grey mouse lemur (*Microcebus murinus*), a nonhuman primate model of brain aging. Touchscreen-based testing of 6 young adults (1–5 years) and 6 old adults (> 7 years) in the procedural rodent dPAL-task revealed significant age-related performance decline, evident in group differences in the percentage of correct decision during learning and the number of sessions needed to reach a predefined criterion. Response pattern analyses suggest decreased susceptibility to relative stimulus-position biases in young animals, facilitating PAL. Additional data from a subset of “overtrained” individuals (n = 7) and challenge sessions using a modified protocol (sPAL) further suggest that learning criteria routinely used in animal studies on PAL can underestimate the endpoint at which a stable performance is reached and that more conservative criteria are needed to improve construct validity of the task. To conclude, this is the first report of an age effect on dPAL and corroborates the role of mouse lemurs as valuable natural nonhuman primate models in aging research.

## Introduction

The cognitive abilities of humans gradually change during aging, with different cognitive functions showing different age-related trajectories (e.g.^1^). Cognitive abilities that predictably decrease during later life, even in healthily aging individuals, are episodic memory and different executive functions^2^. In patients with neurological diseases, functional loss in these and other cognitive domains can be accelerated (e.g.^3^). However, as the risk for many such diseases, as for example Alzheimer’s and Parkinson’s, usually increases with increasing age, it can be difficult to distinguish pathological from healthy cognitive aging^4^.

A neuropsychological tool that has proven valuable in studies on memory decline in healthy and clinical populations and that, today, is also used in drug development programmes and mainstream healthcare is the paired-associates learning (PAL) test of the Cambridge Neuropsychological Test Automated Battery (CANTAB)^5^. In its original version for humans, participants have to memorize and recall the location of visual stimuli presented to them on a computer screen in a trial-unique manner^6^. Therefore, the CANTAB PAL test is mainly considered a test of (episodic) visual object-location memory^5^, though additional cognitive processes are undoubtedly involved (e.g.^7^). In healthy volunteers (age-range 18–90 years), test performance is age-dependent, with performance continuously deteriorating from approximately the 5^th^ decade of life onwards^5^. In addition, the test can differentiate cases of Alzheimer’s disease from healthy controls and patients with depression^8^ and has predictive value for the progression from deficits in memory function insufficient for a neurological diagnosis and mild cognitive impairment to dementia (e.g.^8–10^).

Noticeably, two animal versions of the CANTAB PAL test have been developed, one for monkeys, the monkey PAL^11^, and one for small mammals, the rodent “different PAL” (dPAL)^12^. Comparable to the CANTAB PAL test, both the monkey PAL and the rodent dPAL protocol require the animals to associate visual items with distinct locations on a touchscreen. Different from the CANTAB PAL test, the animal versions usually require repeated training and positive reinforcement for the animals to achieve high performance levels (e.g.^11–15^). Despite this conceptual difference, there is evidence for overlap in the brain areas involved in human CANTAB PAL and rodent dPAL, with a prominent role of hippocampal and medial prefrontal areas in both tasks (for a detailed overview see^5^). In line with this, humans with rare copy number variations in the DLG2 gene showed impaired performance in both, CANTAB PAL^16^ and rodent dPAL^17^, as did homozygous DLG2 knockout mice in the rodent dPAL^17^. Exonic copy number variations in DLG2 have been suggested to be of pathogenic relevance in schizophrenia^18^ and single nucleotide polymorphisms in this gene were linked to Alzheimer’s disease related increases in subcortical shape asymmetry^19^.

Regarding their sensitivity to age-related cognitive decline, little is known for the animal versions of the CANTAB PAL test. For the monkey PAL, there is only one study describing decreased PAL performance in old rhesus macaques (23.0 ± 0.5 years) compared to young individuals (7.1 ± 0.8 years)^14^. For the rodent dPAL, comparable data is missing, because the protocol is usually used to quantify PAL in rather short-lived rodents. Therefore, the first aim of this study was to investigate dPAL performance and its relation to age in a small nonhuman primate, the grey mouse lemur (*Microcebus murinus*). Mouse lemurs are particularly interesting for the investigation of age-effects on dPAL: As primates, they have a closer genetic and physiological distance to humans than rodents. Belonging to the smallest extant primate species, they can readily be trained in the same testing environment as rats without the need for major setup modifications (e.g.^20^). In captivity, mouse lemurs live substantially longer than rodents, with an average life span of about 7 years and maximum ages of up to 18 years^21^. Importantly, the brain of mouse lemurs naturally undergoes biochemical and morphological changes during aging. Biochemical alterations include increased accumulations of amyloid-β (e.g.^22,23^) and tau^24^ proteins as well as iron^25,26^ in the brain of some aged individuals. Morphological changes include age-related, region-specific brain atrophy (e.g.^27–30^) and ventricular expansion (e.g.^22,27,28^). In addition to several reports of age-related cognitive decline in testing environments specifically designed for mouse lemurs (for a concise review see^31^), an age-related decline of object discrimination learning and cognitive flexibility in these primates has been described using a standardized touchscreen task^20^ of which initial acquisition was later found to relate to extracellular cortical accumulations of amyloid-β at death^32^. The general applicability of the rodent dPAL protocol to mouse lemurs has also recently been demonstrated^15^ and the current investigation builds upon this comparative study. Since the behavioural strategies underlying dPAL at high performance levels are the topic of an ongoing debate (e.g.^33–35^), secondary aims of the study were to investigate the relevance of the chosen training criterion and the effect of challenging a subset (n = 7) of highly trained animals in a rodent “same PAL” (sPAL) session.

## Methods

### Ethics declaration

For this study, twelve adult grey mouse lemurs (*Microcebus murinus*) from the breeding colony of the Institute of Zoology of the University of Veterinary Medicine in Hannover (Germany) were trained in a touchscreen-based conditioning environment. Breeding and maintenance of the animals was approved by the Lower Saxony State Office for Consumer Protection and Food Safety (LAVES; reference number: AZ 42,500/1H). The reported, non-invasive experiments were performed in compliance with the German Animal Welfare Act, the NRC Guide for the Care and Use of Laboratory Animals, and the Directive 2010/63/EU of the European Parliament on the protection of animals used for scientific purposes. They were approved by the Animal Welfare Committee of the University of Veterinary Medicine and licensed by LAVES (reference numbers: AZ 33.19-42,502-04-18/3050 and AZ 33.19-42,502-04-14/1454).

### Study animals

The overall sample of this study consisted of 6 young adults (1–5 years at the first day of dPAL) and 6 old adults (7 years or older at the first day of dPAL). Classification as “young” and “old” individuals was chosen to be comparable with a previous touchscreen-based study on age-related cognitive decline in mouse lemurs^20^. Within the age groups, the sex-ratio was balanced (n = 3 per sex and age group). Five of the subjects (three young and two old) were trained in a session-based protocol and some aspects of their training have been reported and discussed in a comparative context (in comparison with rodents and humans), before^15^. The remaining seven subjects (three young and four old) were trained in a home cage-based protocol with 24-h access to the setup on working days (for more details on the training protocols see below).

### Animal housing

Individuals were housed either alone or in groups of two to four animals. Individual home cages (Ebeco Marmoset; width: 0.80 m, depth: 0.65 m, height: 1.55 m) were enriched with wooden sleeping shelters (width: 0.20 m, depth: 0.11 m, height: 0.11 m), hanging pipes, ropes, and tree branches. For group-housed animals, several cages were connected to fit the group size. The temperature in the housing rooms was controlled in a narrow range between 22–27 °C and the relative humidity was set to 55–65%. The artificial day-night-cycle was reversed and changed from a long-day season (LD 14:10; February to September) to a short-day season (LD 10:14; October to January) and back once per year. The diet changed on a regular basis from a mix of seasonal fruits and vegetables on Mondays, Wednesdays, and Fridays to a puree consisting of bananas, rice flakes, and banana milk powder (flakes and milk powder: Milupa Nutricia GmbH; Bad Homburg v. d. H., Germany) on Tuesdays, Thursdays, and the weekend. The puree was additionally supplemented with proteins and vitamins (for a more detailed description of the diet composition see^36^). The weight of the animals was checked on a regular basis (at least once per week). If animals noticeably gained weight during the days of cognitive testing, the diet was temporarily and slightly reduced to compensate the caloric intake through rewards (compare below). Water was always provided ad libitum. Prior to cognitive testing, all individuals underwent an ophthalmological examination to check the functional (e.g. optokinetic nystagmus, general visual object tracking, and pupillary reflexes) and anatomical (lens, ocular pressure, retina) integrity of the animals’ visual apparatus (for methods see^37,38^).

### Cognitive testing

**Figure 1:**
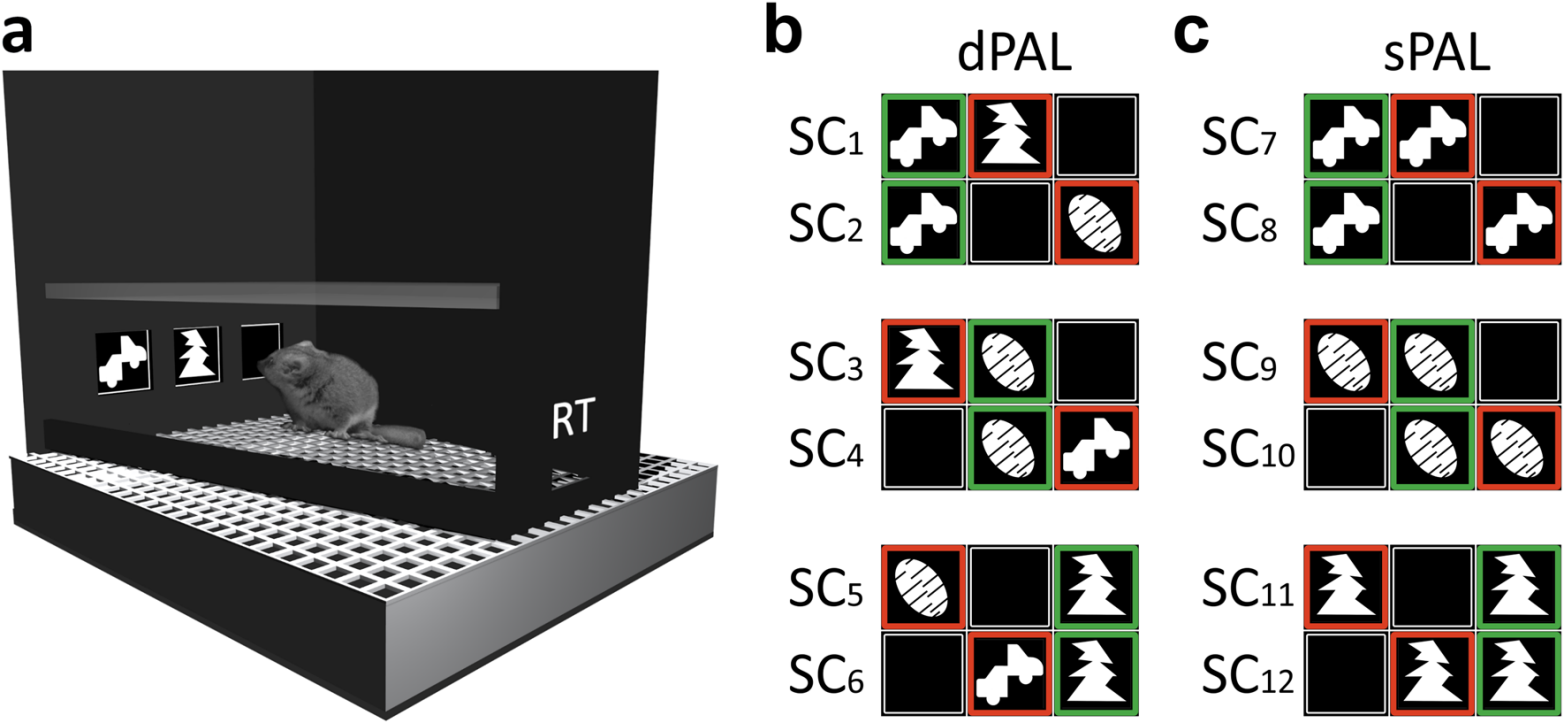
Schematic representations of the setup and the stimulus combination used for cognitive testing. (**a**) Drawing of the trapezoidal (width: front = 245 mm; back = 130 mm; length = 330 mm) testing chamber (left side wall and liquid reward dispenser removed). The touchscreen at the front end was covered by a black Perspex mask with three square (width: 45 mm; height: 45 mm) response windows (left, centre, right). The reward tray (RT) was located at the back end of the chamber. The height of the chamber was limited to 100 mm by a translucent lid. (**b**) Stimulus combinations used during dPAL training (SC_1_–SC_6_). In each of the six possible combinations, one of two different stimuli was presented at its rewarded location. The second, simultaneously presented stimulus was used as a distractor at one of its two unrewarded locations. **c** Stimulus combinations used during the sPAL challenge (SC_7_–SC_12_). In each of the six possible combinations, the same stimulus was presented as doublet, at its rewarded location and, as a distractor, at one of its two unrewarded locations. (**b**, **c**) Green border = S^+^; red border = S^-^. For copyright reasons, the depicted stimuli are those used for the collection of longitudinal data in three individuals. For the first training of all twelve individuals and the subsequent sPAL challenge in seven individuals, the “flower-plane-spider” set of stimuli introduced by Talpos and colleagues^12^ was used. In this original set, the flower was rewarded at the left location, the plane was rewarded at the centre location, and the spider was rewarded at the right location.

In general, the training of the animals took place in customized versions of the Bussey-Saksida touchscreen system (80,604 Touch Screen System for rats, Campden Instruments LTD, Loughborough, England; Fig. 1a) using computer assisted protocols. A detailed description of the training protocols for mouse lemurs is available elsewhere^15,20^. In brief, animals were first trained in a 5-step autoshaping procedure, in which they were conditioned to reliably (more than 80% of the decisions made) interact with random black-and-white stimuli presented behind one out of three possible, pseudo-randomly chosen response windows (RW) on a touchscreen. Afterwards, animals were tested in the CANTAB dPAL protocol (Fig. 1b). During dPAL testing, the animals had to procedurally learn to distinguish three novel pictorial items (a flower, a plane, and a spider) and to associate each of them with a unique, rewarded location (left, centre, right) on the touchscreen. During regular trials, two *different* of these items (hence the “*d*” in dPAL) were presented simultaneously, one (the S^+^) at its rewarded location (flower: left RW; plane: centre RW; spider: right RW), the other one, as a distractor (S^-^), at one of its unrewarded locations. Touch interactions with the S^+^ were rewarded with a small amount of apple juice (15 µl), touch interactions with the S^-^ or with the blank screen behind the third, “empty” RW led to trial termination, signalled by a pure tone (2 kHz, 0.5 s) and a time-out of 5 s. Incorrect trials were followed by correction trials, in which the same stimulus combination as in the preceding trial was reused, until the correct response was eventually given. Correct trials, on the other hand, were followed by the next regular trial. Only responses given during regular trials increased the trial count and were used to assess individual PAL performance. Within a given block of regular trials, each of the six possible stimulus combinations (SC_1_-SC_6_; for examples see Fig. 1b) was presented equally often, using a balanced, pseudo-randomized session design.

Animals trained in the session-based protocol (n = 5) were trained in a single session of 36 trials or a maximum duration of 1 h per day. Session-based training was always conducted within the first 2 h of the artificial dark phase, i.e. at the beginning of the animals’ active phase. Training ended as soon as the animals had reached a criterion of at least 80% correct decisions in two consecutive, complete (= 36 completed trials) sessions (72 trials in total; criterion 1 = C_1_).

Animals trained in the home cage-based protocol (n = 7) had almost unlimited access to the testing chamber on working days. Access was denied only for maintenance purposes (e.g. cleaning of the setup, refilling of the reward depot), which took place once per day during the artificial light phase, i.e. during the inactive phase of the nocturnal animals. Animals could enter the testing chamber directly from their home cage via a short tunnel. The general testing procedure was the same as for the animals trained in the session-based procedure. The only difference was that the number of trials an individual could complete per day was only theoretically limited by the size of the reward depot holding 2000 rewards (30 ml). While this limit was never reached, animals trained in the home cage-based protocol usually completed substantially more than 36 trials per day. The average number of trials per day during training to C_1_ was 122 and increased with increasing training. To exploit this advantage of the procedure and to allow comparisons of different criteria, individuals were trained to a more conservative criterion of at least 80% correct decisions in a block of 360 trials (criterion 2 = C_2_). Once this criterion was reached, animals were submitted to a 120-trials sPAL challenge (compare^34,35^ for the utilisation of the sPAL challenge in rodents). During this challenge, the designated S^+^ of a given stimulus combination remained the same item-location match as in the dPAL. The distractor stimulus (S^-^; item-location mismatch), however, was the *same* item (hence the “*s*” in sPAL) as the S^+^ (SC_7_-SC_12_; for examples see Fig. 1c).

### Longitudinal testing

Three male individuals from this study, which were originally tested at young age, were retested using the home cage-based protocol and a novel set of stimuli (Fig. 1; high resolution bitmaps of these stimuli are provided in the supporting materials as stimulus templates 1–3). One individual was retested immediately after finishing the original training to C_2_ (Δ_age_ between the first day of the original testing and the first day of retesting = 2 months). The second individual was retested approximately 3 years (Δ_age_ = 37 months) after the original training to C_2_. The third individual was retested approximately 5.5 years (Δ_age_ = 67 months) after the original training to C_1_ (originally trained in the session-based protocol). Before retesting, all three individuals had to repass the ophthalmological examination as well as the autoshaping procedure (see above). Since the number of subjects that were tested longitudinally did not suffice for inferential statistics, results are presented as supplementary material (Fig. S1).

### Data analyses

To allow the investigation of potential age-effects on dPAL in the pooled data set from all twelve individuals tested, different approaches for the quantification of the learning performance were taken. In a session-independent approach, the cumulative number of correct decisions during regular trials was calculated for the first 2160 trials (corresponding to 60 sessions of 36 trials per session) of each individual. In a second approach, the continuous performance data from each individual was divided into a sequence of separate sessions of 36 trials, for which individual percentages of correct responses could be calculated. Using this data, the number of sessions needed to reach C_1_ could also be quantified for the individuals trained in the home cage-based protocol. A similar approach of dividing the continuous data into discrete blocks of 5 sessions (= 180 trials) was used to calculate learning curves and for the analysis of individual response patterns during dPAL. For all additional analyses, only data from the home cage trained subsample were used.

### Descriptive statistics

**Figure 2:**
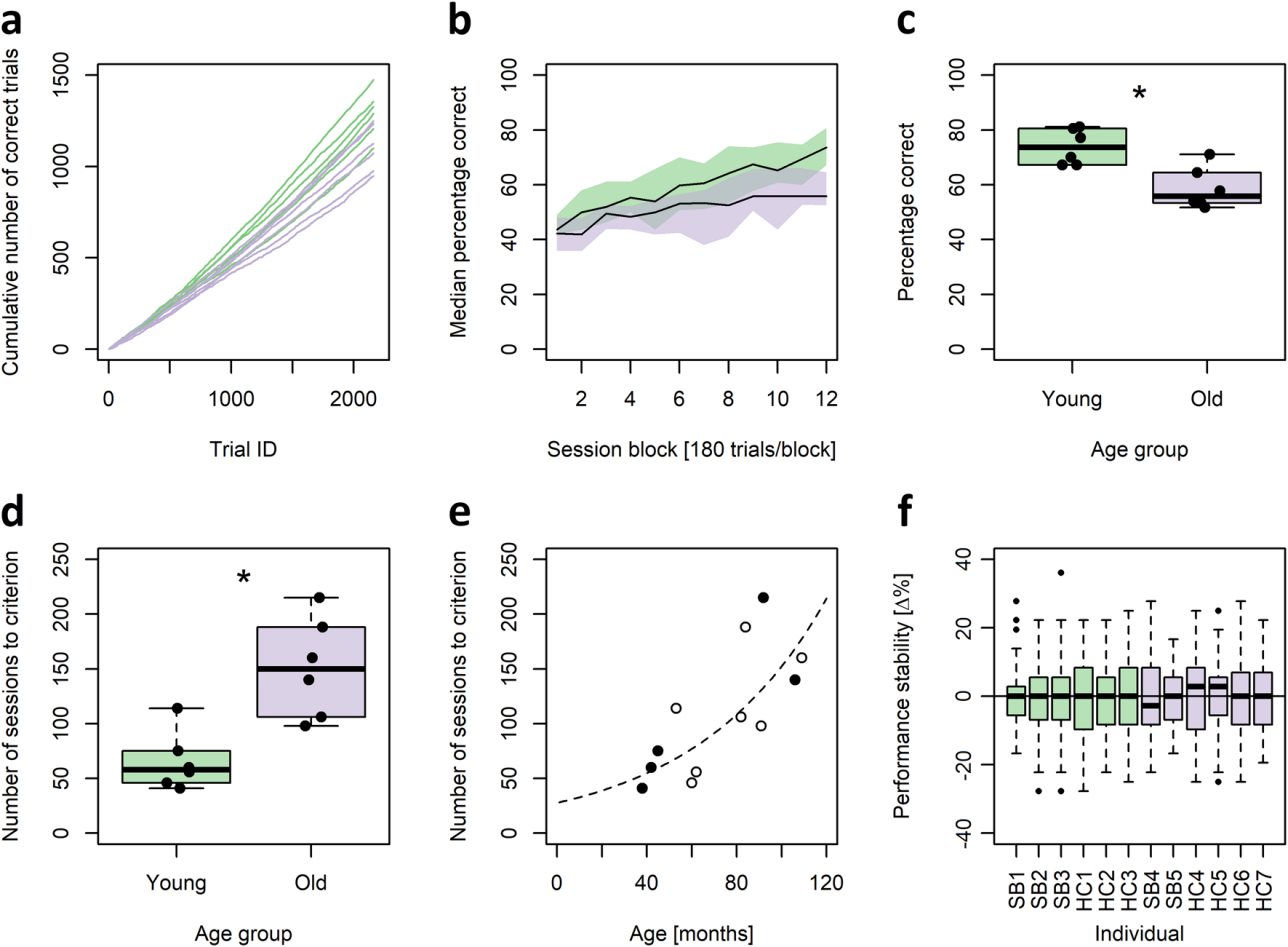
PAL performance and age. (**a**) Cumulative sums of correct responses for young (green) and old (purple) individuals over the first 2160 trials (60 sessions). (**b**) Median learning curves for young and old individuals. Shaded green (young) and purple (old) areas represent the 95% empirical bootstrap confidence intervals of the respective medians. (**c**, **d**) Group-level comparisons of the individual percentages of correct decisions at session block 12 (**c**) and the number of sessions needed to reach learning criterion C_1_ (**d**). Significance code: *p < 0.025; exact Wilcoxon rank sum tests. (**e**) Number of sessions to C_1_ as a function of age in months. Filled circles represent individuals trained in a session-based protocol (SB_1_–SB_5_), hollow circles represent individuals trained in a home cage-based protocol (HC_1_–HC_7_). The dashed line represents an exponential fit to the data. (**f**) Individual session-to-session performance stabilities calculated for the first 60 sessions (n = 59). Young individuals are represented in green, old individuals in purple.

Due to the small sample sizes, only nonparametric statistics were used. Accordingly, in the main text, data sets are summarized by presenting medians as measure of centre as well as minima and maxima to describe variability. In the figures, group data is usually presented as boxplots, with horizontal black lines representing the sample median, the box representing the inter-quartile-range (IQR), and whiskers extending to the most extreme data points within a distance of 1.5 times the IQR from the boundaries of the box. Data points outside this range are depicted as potential outliers. In one case (Fig. 2b) median learning curves are presented. For these learning curves, 95% empirical bootstrap confidence intervals were calculated using 1000 bootstrap iterations with replacement per median.

### Inductive statistics

To compare measured variables between young and aged individuals, two-tailed exact Wilcoxon rank sum tests were used at an alpha-level of 0.05. When multiple similar variables were compared between age-groups, i.e. (i) “the percentage of correct responses in session block 12” and “the number of sessions to C_1_“ as well as (ii) the bias indices for “relative stimulus position”, “absolute RW location”, and “item”, the alpha-level was reduced to (i) 0.025 (= 0.05/2) and (ii) 0.016 (= 0.05/3), respectively. To estimate the effects of age in months and the training method used on learning performance, the number of sessions to C_1_ was modelled as a linear function of both, age and method. To confirm the age-effect on learning performance suggested by this parametric modelling approach, a nonparametric, two-tailed Spearman correlation analysis was calculated at an alpha-level of 0.05. To compare the performance of the home cage trained individuals before and during the sPAL challenge, a two-tailed, paired exact Wilcoxon signed rank test at an alpha-level of 0.05 was used. Finally, to identify session blocks with response bias (preference *or* avoidance) in the individual response pattern analyses or *over*representations of a certain error type during the sPAL challenges in the individual error pattern analyses, *two*-tailed and *one*-tailed binomial tests at an alpha of 0.05 were used, respectively. All statistical analyses were performed in R^39^.

## Results

### Age effects on early task performance

The cumulative number of correct responses after 60 sessions of 36 trials each, which was the maximum number of sessions available for all twelve subjects tested, varied between a minimum of 1100 and a maximum of 1474 (median = 1308) in the young adults (n = 6). In the aged adults (n = 6) it varied between a minimum of 948 and a maximum of 1249 (median = 1099; Fig. 2a). Plotting the individual cumulative numbers of correct responses against the trial count suggests a clear performance split between young and aged adults (Fig. 2a). This impression is confirmed when the median percentage of correct responses in successive blocks of 5 sessions (180 trials) is compared between young and aged adults (Fig. 2b). The median learning curve of the young adults constantly (for session block 1–12) lies above the median learning curve of the aged adults with a separation of the 95% empirical bootstrap confidence intervals (n_boot_ = 1000) of the group medians at session block 12 (Fig. 2b). Indeed, the percentage of correct responses in block 12 (sessions 56–60) is significantly higher in the young adults compared to the aged adults (two-tailed exact Wilcoxon rank sum test, n_young_ = 6, n_aged_ = 6, W = 33, *p *value = 0.013, alpha = 0.025; Fig. 2c).

### Age effects on task acquisition (sessions to criterion 1)

The number of sessions (36 trials per session) needed to reach a designated learning criterion of at least 80% correct choices in two consecutive sessions (C_1_), as an often-used performance measurement in dPAL and similar tasks, also differed significantly between young and aged adults (two-tailed exact Wilcoxon rank sum test, n_young_ = 6, n_aged_ = 6, W = 2, *p* value = 0.008, alpha = 0.025; Fig. 2d). For young adults, the number of sessions to C_1_ varied between a minimum of 41 and a maximum of 114 sessions (median = 58 sessions; Fig. 2d). For aged adults, it varied between a minimum of 98 and a maximum of 215 sessions (median = 150 sessions; Fig. 2d). Modelling the number of sessions needed to C_1_ as a function of age (in months) and the method used (session-based training *vs.* home cage training) demonstrated a significant link between the number of sessions needed to C_1_ and age (linear model, n = 12, df = 9, Estimate = 1.788, SE = 0.517, t = 3.46, *p* = 0.007, alpha = 0.05) but not between the number of sessions needed to C_1_ and the training method used (linear model, n = 12, df = 9, estimate =  − 19.163 , SE = 25.167, t =  − 0.761, *p* = 0.466). When modelling the number of sessions needed to C_1_ as a function of age alone, an exponential model provided the better fit (Fig. 2e), as the linear model predicted negative session numbers at very young ages. A nonparametric Spearman analysis confirmed a highly significant positive correlation between the age in months and the number of sessions needed to C_1_ (two-tailed Spearman’s correlation; n = 12, rho = 0.748, S = 72, *p* = 0.007, alpha = 0.05; Fig. 2e).

### Possible factors underlying the found age differences in dPAL

**Figure 3:**
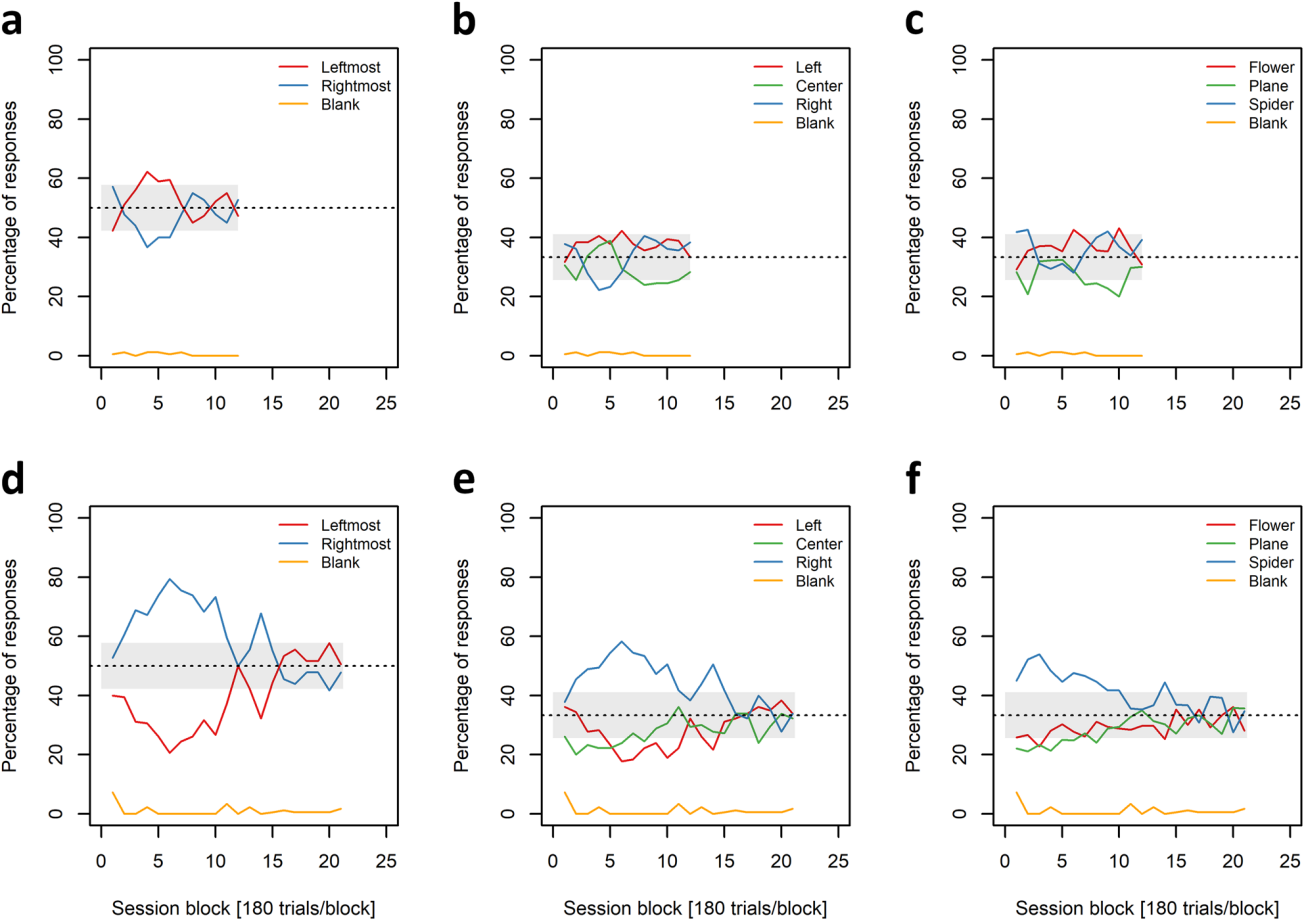
Exemplary response profiles of a young (SB_1_; **a**–**c**) and an old (HC_7_; **d**–**f**) individual. (**a**–**f**) Percentages are presented for session blocks of 5 sessions (180 trials). Dashed black lines represent the expected response probabilities for the presented instances, shaded grey areas include all responses for which the probability is > 5% (as based on two-tailed binomial tests: n = 180, alpha = 0.05). Session blocks in which this area was left were considered as biased for the represented instances. (**a**, **d**) Individual percentages of responses made to either the leftmost or the rightmost of the two simultaneously presented items (relative position). (**b**, **e**) Individual percentages of responses made to the stimulus presented in either the left RW, the centre RW, or the right RW (absolute RW location). (**c**, **f**) Individual percentages of responses made to either the flower, the plane, or the spider (item).

As C_1_ demanded a performance of above 80% correct choices in two consecutive sessions, individuals with high session-to-session performance fluctuations would have been at a disadvantage to reach C_1_. Comparing the individual measures of centre (median) and variability (interquartile-range = IQR) of session-to-session performance fluctuations over the first 60 sessions between the age-groups, however, did not reveal any significant differences: The individual medians of both groups centred around zero and the individual IQRs in both groups were not significantly different (two-tailed exact Wilcoxon rank sum test, n_young_ = 6, n_aged_ = 6, W = 14.5, *p* value = 0.619, alpha = 0.05; Fig. 2f). In addition, it was investigated whether the decisions made by an individual in a given block of 5 sessions (180 trials) were biased towards (= preference) or away from (= aversion) a relative stimulus position (leftmost or rightmost), an absolute RW location (left RW, center RW, or right RW), or one of the two simultaneously presented items (flower, plane, or spider). A session block was considered biased, if the number of responses to any of these instances differed significantly (two-tailed binomial tests, n = 180, alpha = 0.05) from the expected response probabilities (relative stimulus position: p_exp_ = 0.5; RW location or item: p_exp_ = 0.333). All twelve subjects, from the fastest to the slowest learner, showed at least one type of bias in the first block, which means at the very beginning of training (Fig. 3 and Figs. S2–S13). In addition, all twelve individuals developed and overcame further biases/response strategies in the course of training to C_1_ (Fig. 3 and Figs. S2–S13). Based on the categorization of session blocks as being biased or not, individual bias indices (BI) were calculated for each investigated category (relative stimulus position, absolute RW location, and item) as the fraction of the individual session blocks to C_1_ that showed at least one of the biases of the respective category. Comparing these indices between young and aged adults revealed significantly reduced indices for relative location biases in young individuals (two-tailed exact Wilcoxon rank sum test, n_young_ = 6, n_aged_ = 6, W = 1.5, *p* = 0.006, alpha = 0.016; supporting Fig. S14a). For the other indices (absolute RW location and item), differences between the age groups were not significant (two-tailed exact Wilcoxon rank sum test, n_young_ = 6, n_aged_ = 6, W ≥ 13, *p* ≥ 0.48, alpha = 0.016; supporting Fig. S14b,c).

### Suitability of C_1_ as a learning criterion

**Figure 4:**
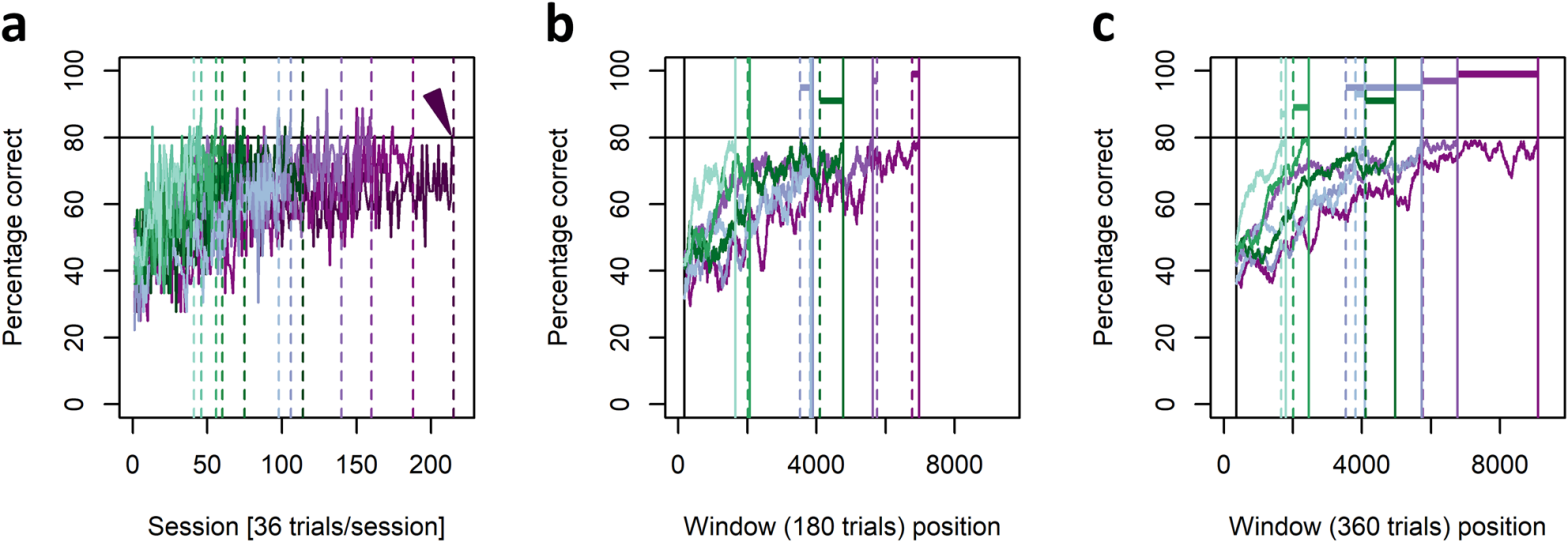
Individual learning curves and criterion quality. (**a**–**c**) Green colours represent young individuals, blue and purple colours represent aged individuals. (**a**) Individual learning curves to C_1_ from all tested individuals (n = 12). Solid horizontal black line: desired criterion performance; Dashed vertical lines: sessions at which C_1_ was reached by the respective individual. The arrowhead indicates for one example that C_1_ had sometimes been reached before the individuals’ performances stabilised at 80%. (**b**, **c**) Individual learning curves using rolling criteria of 80% correct responses in a window of (**b**) 180 trials and (**c**) 360 trials (= C_2_) and data from the overtrained individuals (n = 7). As before, dashed vertical lines indicate the point at which C_1_ was reached, solid vertical lines indicate the point at which the rolling criterion was reached by the respective individual. Coloured horizontal lines illustrate the difference in trials needed to C_1_ and the depicted rolling criterion for each individual. (**b**) C_1_ and the rolling criterion are in good agreement. (**c**) The individual numbers of trials to C_1_ and C_2_ can differ substantially, especially in slow learners.

A closer examination of the individual learning curves to C_1_ suggested that, in some individuals, C_1_ had not reliably detected the onset of stable task performance but had rather underestimated achievement of the actual dPAL target. As individual percentages of correct decisions were calculated from sessions of only 36 trials, these performance estimates were vulnerable to stochastic noise and some individuals crossed the designated threshold of 80% correct decisions in two consecutive sessions before their performance had actually plateaued at 80% or above (Fig. 4a). Therefore, the home cage trained individuals (n = 7) were (over-) trained to a second, rolling criterion of 80% or more correct choices in a window of 360 successive trials (C_2_), which loosely translates to an average performance of 80% correct choices in 10 consecutive sessions of 36 trials (Fig. 4c). This additional training data was used to compare C_1_ to different simulated rolling criteria with window sizes ranging from 72 to 360 trials (for two example comparisons with window sizes of 180 and 360 trials see Fig. 4b,c; for comparisons with all simulated rolling criteria from 72 to 360 trials in incremental steps of 18 trials see supporting Video S1). This qualitative comparison revealed that C_1_ is in good agreement (only small differences) with rolling criteria ranging from 144 to 180 trials in window size, but that C_1_ clearly underestimates dPAL in some animals if more stable task performances are requested (window sizes of 288 trials and above). Despite the underestimation of task acquisition (i.e. the endpoint of asymptotic performance) in some animals by C_1_, the relative learning durations, i.e. individual ranks on a scale from the slowest to the fastest learner, were quite similar at C_1_, C_2_, and the simulated steps in between, with animals occasionally changing ranks at the centre positions, but not the extremes (Fig. 4, Video S1).

### Error patterns during sPAL challenge sessions

**Figure 5:**
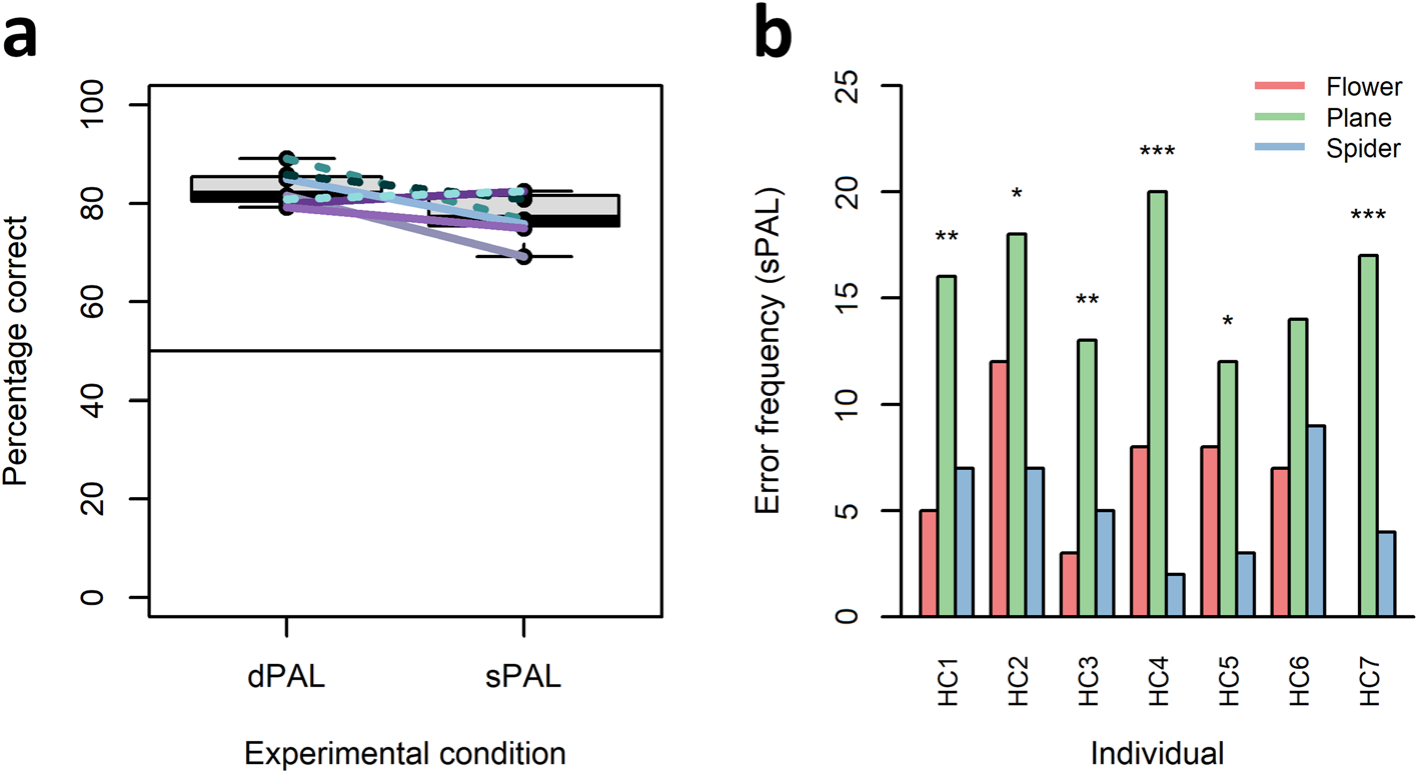
Performance and error patterns during sPAL challenge sessions. (**a**) Comparison of individual performances directly before (last 120 trials in dPAL) and during the sPAL challenge (120 trials). Dashed and solid horizontal lines represent individual trajectories. (**b**) Individual error frequencies during sPAL additionally separated by the identity of the S^+^ (flower = SC_7_ + SC_8_; plane = SC_9_ + SC_10_; spider = SC_11_ + SC_12_). Significance code: *p < 0.05, **p < 0.01, ***p < 0.001; one-tailed Binomial tests (for details see supporting Table S1).

Once C_2_ had been reached, the seven overtrained individuals were additionally challenged with a modified protocol, the sPAL task. In this task, the distractor stimulus of a given trial, i.e. the item presented at an incorrect location, was the same item as the respective S^+^. Therefore, response strategies based on learned item constellations or sequences inevitably fail in the sPAL task. To avoid that the animals learn during the challenge, sPAL sessions were limited to 120 trials. Individual sPAL performance was compared to the performance during the last 120 trials completed in the dPAL protocol. During the last 120 trials of dPAL, individual percentages correct varied between a minimum of 79.17% and a maximum of 89.17% (median = 81.67%). During the sPAL challenges, individual percentages correct varied between a minimum of 69.17% and a maximum of 82.5% (median = 76.67%; Fig. 5a). There was no significant difference between individual dPAL and sPAL performances (two-tailed, paired exact Wilcoxon signed rank test, n = 7, V = 25, *p* = 0.078, alpha = 0.05; Fig. 5a). While five individuals showed a moderate performance decline in the sPAL challenge, two showed a small performance increase in the sPAL challenge when compared to their final dPAL performance. The individual error profiles during sPAL were highly homogeneous, with a significant overrepresentation (one-tailed binomial tests, p_exp_ = 0.33, alpha = 0.05; for individual statistics see supporting Table S1) of errors in those trials in which the S^+^ was the stimulus with its rewarded location at the centre position (SC_9_ and SC_10_; Fig. 5b) in six of the seven individuals (and a trend towards a similar pattern in the seventh animal; supporting Table S1).

## Discussion

This study provides detailed performance data from the touchscreen-based rodent dPAL protocol applied to a nonhuman primate model in aging-research, the grey mouse lemur. The first major finding of the study was significant age effects on task acquisition with aged individuals of 7 years and older presenting lower percentages of correct decisions after a fixed number of trials than younger individuals. In addition, aged individuals needed significantly more sessions to reach a commonly used learning criterion (C_1_) than younger individuals and the individual number of sessions to C_1_ showed a highly significant positive correlation with age. To my best knowledge, this is the first description of age effects in dPAL. In previous mouse lemur studies on cognitive aging in other domains, it was often found that some aged individuals perform just as well as young adults, while others show a clear performance decline. In these studies, a high performance variance in aged subpopulations was usually attributed to a possible heterogeneity of this subpopulation, consisting of individuals that aged healthily along animals that aged pathologically (e.g.^20,29,40^). In dPAL, on the other hand, there was an almost full separation of acquisition performance between young and aged individuals. Thus, visuo-spatial PAL performance may quite strongly relate to age, even in healthily aging individuals. A similarly strong performance separation between young and aged mouse lemurs has previously been found in a spatial reversal task^29^ and one could speculate that the necessity for spatial processing both tasks have in common plays a role in their sensitivity for age-related cognitive decline.

In the analyses of possible factors underlying the slowed visuo-spatial PAL acquisition in aged individuals, the second major finding of the study was that young and aged adults did not significantly differ in their session-to-session performance stability or in their susceptibility to item or absolute RW location biases, both occurring regularly in similar fractions in both groups. They did, however, differ in their susceptibility to relative stimulus position biases. In aged individuals, fractions of relative stimulus location biased sessions were comparable to those biased for absolute RW location and item. Young individuals showed a significantly reduced susceptibility to relative stimulus position biases compared to aged individuals. Since the leftmost and the rightmost stimulus were rewarded in 50% of the trials each, application of a response strategy based on relative stimulus position drives performance towards chance level (as do response strategies based on absolute RW location or item identity), which means away from the learning criterion. It is, thus, likely that more frequent and/or more stable utilisation of response strategies based on relative stimulus position in aged individuals rather than a strategy based on the recall of visuo-spatial item-location paired-associates partially contributed to the observed age effect on task acquisition. Interestingly, more frequent strategy shifts and higher strategic conservatism in favour for a scanning rather than a recall-based strategy in aged adults compared to young adults have been described for humans in a conceptually related noun pair matching task^41,42^ and strategic components have also been discussed to contribute to age-related performance decline in visuo-spatial CANTAB PAL in humans^7^.

An additional finding of the individual response pattern analyses was the observation of generally high dynamics of individual response strategies, especially during early training. As described, all subjects used at least one of the more elemental response strategies (relative stimulus position, absolute RW location, item) in session block 1 and regularly switched or mixed these task irrelevant strategies during training to criterion. In previous studies on dPAL in animals and humans, two main strategies were usually discussed to underlie high accuracy performance in this task, a non-spatial strategy based on the sequence of the individual items or the overall appearance of a given stimulus combination and a spatial strategy based on item-location paired-associates (e.g.^15,34,35^). The here-presented analyses demonstrate that more elemental response strategies, based on stimulus identity, absolute RW location, or relative item position, dominate initial acquisition of the dPAL task in mouse lemurs. In accordance with previous discussions, at C_1_, these elemental strategies had usually been abandoned (Figs. S2–S13), most likely in favour for a more effective strategy based on the recall of visuo-spatial paired-associates (see below; compare^15^). This finding is in line with data from lesioning studies in rodents exploring the neural systems involved in dPAL: In mice, excitotoxic lesioning of the dorsal striatum almost completely prevented performance increase during initial dPAL (tracked over 40 sessions of 36 trials, corresponding to a maximum of 1440 trials), while hippocampal excitotoxic lesioning of the hippocampus had no significant effect on initial task acquisition^33^. However, when tracked over considerably more trials (3000 +) and until asymptotic performance was reached, a different study in mice could demonstrate slowed acquisition of the dPAL task after excitotoxic lesioning of the dorsal hippocampus^34^. In rats, acquisition of the dPAL task was additionally shown to be sensitive to excitotoxic lesioning of the medial prefrontal cortex^35^. The dorsal striatum, thus, may be especially important during early task acquisition, when animals, like the mouse lemurs of the present study, tend to use and alternate between more elemental, task irrelevant response strategies (compare^35^ for a highly similar observation in mice). At later stages of task acquisition, which means towards reaching a high-level, asymptotic performance, these elemental strategies are gradually replaced by a more complex strategy involving hippocampus-dependent visuo-spatial processing. The medial prefrontal cortex contributes to this transition, likely because of its roles in object-location learning and memory (e.g.^43,44^) and/or strategy switching (e.g.^45^). Post-acquisition performance in the dPAL task has been found to be sensitive to pharmacological manipulation or lesioning of the dorsal hippocampus in mice and rats^12,34^, suggesting that a strategically homogeneous, hippocampus-dependent endpoint is reached at asymptotic performance.

To estimate this endpoint of task acquisition in dPAL and similar touchscreen- and session-based protocols, it is recommended to use a criterion of more than 80% correct decisions in two consecutive sessions^46^. Accordingly, we used this criterion to quantify learning rates in touchscreen-based pairwise discrimination and reversal learning in mouse lemurs^20^ and in our original comparative study on dPAL in mouse lemurs and humans^15^. The final major finding of the current study, resulting from a simulation of different learning criteria in the home cage trained individuals, is that this criterion (C_1_) underestimates the point at which asymptotic performance is reached in some individuals. Relative learning durations were similarly (though not identically) assessed by all simulated criteria from C_1_ to C_2_. This suggests that conservative criteria, such as C_2_, should preferentially be used for comparisons of relative learning dynamics, but that less conservative criteria, such as C_1_, may be used as an alternative in studies in which the study duration is limited by important factors, such as the life expectancy of a species or a disease model. For studies in which the determination of stable performance and strategic homogeneity is vital, as post-acquisition phenomena are of interest (e.g. in studies on task performance retention after local lesioning), more conservative criteria should mandatorily be used. This is especially true for species in which trial numbers are small if session-based dPAL training is applied, such as mice and mouse lemurs, where individual sessions usually comprise a maximum of 36 trials (e.g.^15,33,47,48^). That C_2_, which means a rolling criterion of 80% correct choices in a window of 360 trials, may serve as such a conservative learning criterion for the detection of strategically more homogeneous endpoints of dPAL in mouse lemurs is supported by the presented sPAL challenge performance data: While the median performance in the sPAL challenge dropped by 5% compared to pre-challenge dPAL performance, this was not a significant difference in the success rates between both testing conditions. Also, despite the slight decrease, sPAL performance was far from dropping to chance level, which would have been expected if animals had strictly used a non-spatial strategy based on the sequence of the individual items or the overall appearance of a given stimulus combination at C_2_. Comparable results from sPAL challenges in rodents were taken as evidence for the utilization of a spatial strategy based on item-location paired-associates (e.g.^34,35^) during pre-challenge dPAL and the sPAL challenge.

High strategic uniformity after dPAL acquisition to C_2_ is further evident in the presented sPAL error profile analysis. A generally increased error rate in the stimulus combination set consisting of SC_9_ and SC_10_ (Fig. 1) suggests that these stimulus combinations were particularly challenging for all subjects tested in sPAL and that the spatial strategy based on item-location paired-associates was more readily applied to the remaining sets (SC_7_ + SC_8_, SC_11_ + SC_12_). As discussed for a comparable analysis before^15^, stimulus combination set SC_9_ + SC_10_ consists of two stimulus combinations with close spatial proximity of the presented items and, therefore, puts higher demands on spatial pattern separation than the other stimulus combination sets (SC_7_ + SC_8_, SC_11_ + SC_12_) with only one such constellation per set (Fig. 1). This may explain the higher difficulty all animals had with stimulus combination set SC_9_ + SC_10_ of the sPAL protocol. Taken together, the observed sPAL performance data and individual error profiles support the postulation that C_2_ reliably assessed a uniform endpoint of learning in dPAL.

## Conclusion

The here-presented data on PAL in mouse-lemurs and our previous comparative study strongly suggest that conserved cognitive mechanisms underlie dPAL performance in animals from rodents to primates: While task acquisition rates have already been shown to be highly similar in young mouse lemurs, rats, and mice^15^, the here-presented data demonstrates that acquisition of the dPAL task in mouse lemurs is highly complex, involving the establishment and rejection of elemental response strategies at early stages and the dominance of a spatial strategy based on the recall of visuo-spatial paired-associates at asymptotic performance. Highly similar results have been reported for rodents (e.g.^35^), supporting the idea of parallel cognitive processes being involved across species. In rodents, these processes have so far been identified as striatal processing^33^, likely due to the procedural nature of the task and the involvement of elemental response strategies, as well as hippocampal and medial prefrontal processing (e.g.^34,35^), likely due to the necessity to establish and recall object-location paired associates and to abandon more elemental strategies at late stages of the task. With the hippocampus and medial prefrontal cortex being involved, the dPAL protocol shares important neuronal substrates with the human version of the task (CANTAB PAL), suggesting a high translational potential of dPAL (compare^5^), if the endpoint of task acquisition is correctly identified.

Most importantly, the here-presented data provides first evidence for a natural age-effect on learning dynamics in dPAL in a cross-sectional sample of twelve nonhuman primates. Slowed learning in aged mouse lemurs may mainly reflect impairments in spatial processing, as it has also been demonstrated in other spatial paradigms^29^, but individual response patterns suggest that higher strategic conservatism in aged individuals likely contributes to the effect. Given that similar results have been found in conceptually related tasks in humans (e.g.^7,41^) and that the dPAL protocol has also successfully been translated to humans^15,17^, it would be highly interesting to investigate possible age-effects on dPAL in humans. Overall, the present study corroborates the high value of the dPAL protocol in comparative cognitive research and that of mouse lemurs as natural models in research on cognitive aging.

## Supplementary Information

Supplementary Information 1.

Supplementary Information 2.

Supplementary Information 3.

Supplementary Information 4.

Supplementary Information 5.

Supplementary Information 6.

## Data Availability

All data will be made available upon reasonable requests.
