# Supplementary material for: Age affects procedural paired-associates learning in the grey mouse lemur (Microcebus murinus)
Source: Sci Rep. 2021 Jan 13;11:1252. doi: 10.1038/s41598-021-80960-y (PMC7806666; doi:10.1038/s41598-021-80960-y)
Supplement: Supplementary file 1 — Supplementary Information 1. [file 41598_2021_80960_MOESM1_ESM.pdf]

Age affects procedural paired-associates learning in the grey mouse lemur (*Microcebus murinus*)

Daniel Schmidtke[1]

[1]Institute of Zoology, University of Veterinary Medicine Hannover, Hannover, Germany

Supporting figures

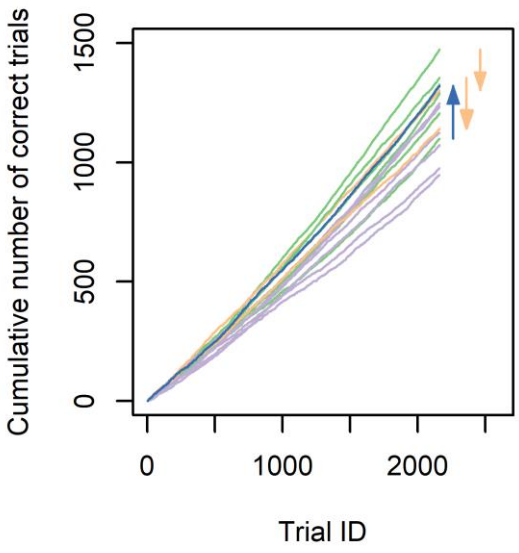

Fig. S1. **Longitudinal data.** Cumulative sums of correct responses for young (green) and old (purple) individuals over the first 2160 trials (60 sessions). The blue line represents the second cumulative sum of a young adult that was tested two-times in a row (Δ_{age} = 2 months) on two different stimulus sets. Orange lines represent the second cumulative sums of two previously young adults that were retested at old age (Δ_{age} = 37 & 67 months). Arrows indicate the size of the performance differences at trial 2160 between initial testing (base of the arrow) and retesting (tip). In the young male individual tested two times in a row, the cumulative number of correct responses after 60 sessions increased between tests from 1100 to 1322. In the two male individuals tested at young and at old age, the cumulative number of correct responses after 60 sessions dropped between tests from 1474 to 1303 (Δ_{age} = 37 months) and from 1356 to 1141 (Δ_{age} = 67 months). The fact that the individual tested twice at young age showed a performance increase, may be indicative for a training effect. An immediate “rule transfer” from the first set of stimuli to the second, however, could not be observed, as all longitudinally tested individuals started the second training at around chance-level performance.

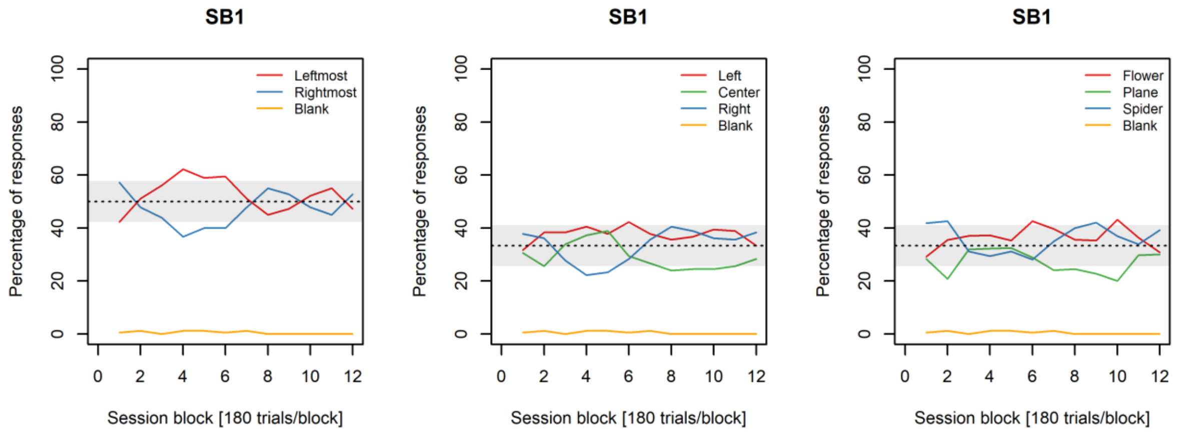

Fig. S2. **Response profiles of young female individual SB_1 during session-based training to C_1.** Percentages are presented for session blocks of 5 sessions (180 trials). Dashed black lines represent the expected response probabilities for the presented instances, shaded grey areas include all responses for which the probability is >5% (as based on two-tailed binomial tests: n = 180, alpha = 0.05). Session blocks in which this area was left were considered as biased for the represented instances. **Left:** Individual percentages of responses made to either the leftmost or the rightmost of the two simultaneously presented items (relative position). **Centre:** Individual percentages of responses made to the stimulus presented in either the left RW, the centre RW, or the right RW (absolute RW location). **Right:** Individual percentages of responses made to either the flower, the plane, or the spider (item).

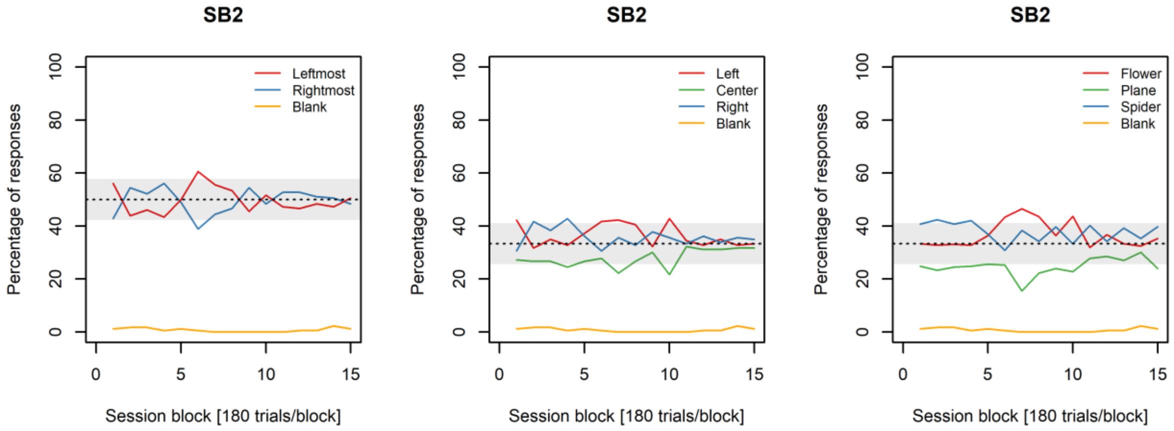

Fig. S3. **Response profiles of young female individual SB_2 during session-based training to C_1.** Percentages are presented for session blocks of 5 sessions (180 trials). Dashed black lines represent the expected response probabilities for the presented instances, shaded grey areas include all responses for which the probability is >5% (as based on two-tailed binomial tests: n = 180, alpha = 0.05). Session blocks in which this area was left were considered as biased for the represented instances. **Left:** Individual percentages of responses made to either the leftmost or the rightmost of the two simultaneously presented items (relative position). **Centre:** Individual percentages of responses made to the stimulus presented in either the left RW, the centre RW, or the right RW (absolute RW location). **Right:** Individual percentages of responses made to either the flower, the plane, or the spider (item).

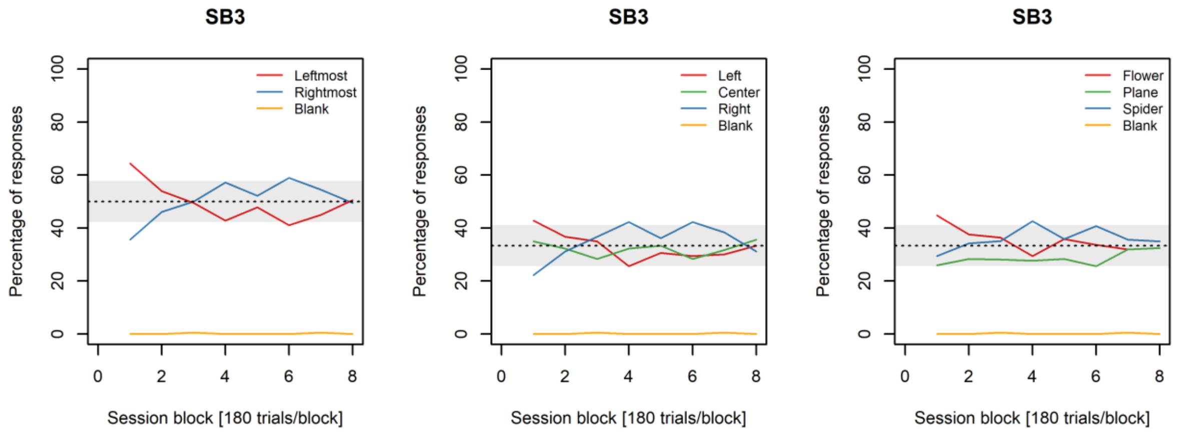

Fig. S4. **Response profiles of young male individual SB_3 during session-based training to C_1.** Percentages are presented for session blocks of 5 sessions (180 trials). Dashed black lines represent the expected response probabilities for the presented instances, shaded grey areas include all responses for which the probability is >5% (as based on two-tailed binomial tests: n = 180, alpha = 0.05). Session blocks in which this area was left were considered as biased for the represented instances. **Left:** Individual percentages of responses made to either the leftmost or the rightmost of the two simultaneously presented items (relative position). **Centre:** Individual percentages of responses made to the stimulus presented in either the left RW, the centre RW, or the right RW (absolute RW location). **Right:** Individual percentages of responses made to either the flower, the plane, or the spider (item).

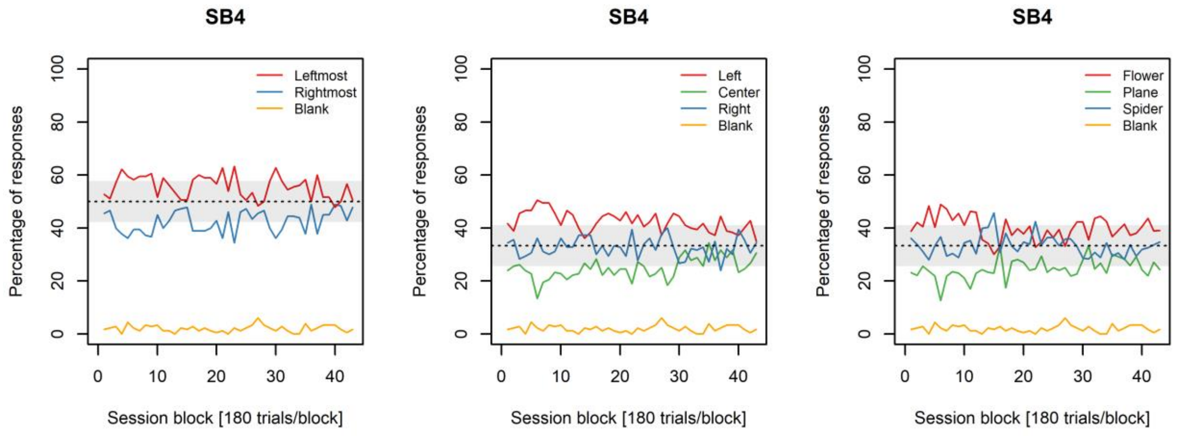

Fig. S5. **Response profiles of old female individual SB_4 during session-based training to C_1.** Percentages are presented for session blocks of 5 sessions (180 trials). Dashed black lines represent the expected response probabilities for the presented instances, shaded grey areas include all responses for which the probability is >5% (as based on two-tailed binomial tests: n = 180, alpha = 0.05). Session blocks in which this area was left were considered as biased for the represented instances. **Left:** Individual percentages of responses made to either the leftmost or the rightmost of the two simultaneously presented items (relative position). **Centre:** Individual percentages of responses made to the stimulus presented in either the left RW, the centre RW, or the right RW (absolute RW location). **Right:** Individual percentages of responses made to either the flower, the plane, or the spider (item).

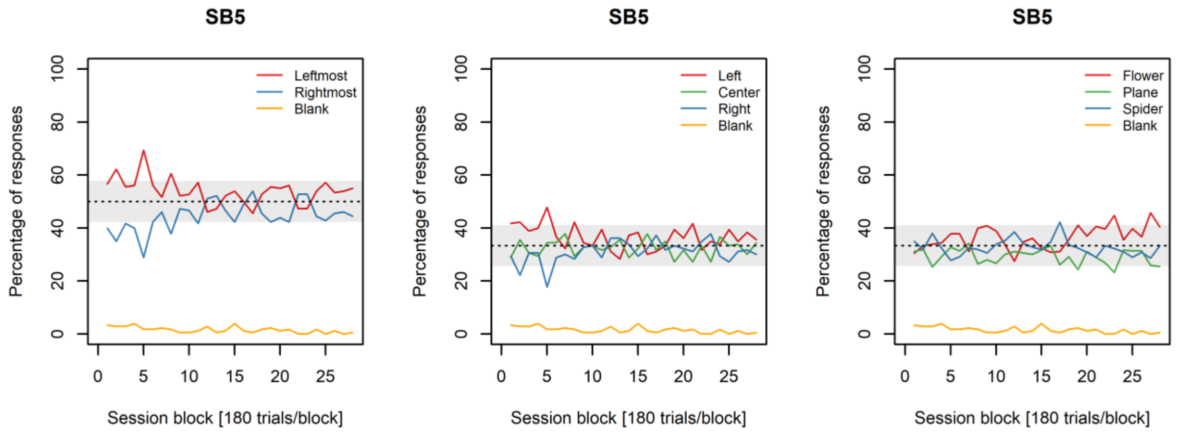

Fig. S6. **Response profiles of old female individual SB_5 during session-based training to C_1.** Percentages are presented for session blocks of 5 sessions (180 trials). Dashed black lines represent the expected response probabilities for the presented instances, shaded grey areas include all responses for which the probability is >5% (as based on two-tailed binomial tests: n = 180, alpha = 0.05). Session blocks in which this area was left were considered as biased for the represented instances. **Left:** Individual percentages of responses made to either the leftmost or the rightmost of the two simultaneously presented items (relative position). **Centre:** Individual percentages of responses made to the stimulus presented in either the left RW, the centre RW, or the right RW (absolute RW location). **Right:** Individual percentages of responses made to either the flower, the plane, or the spider (item).

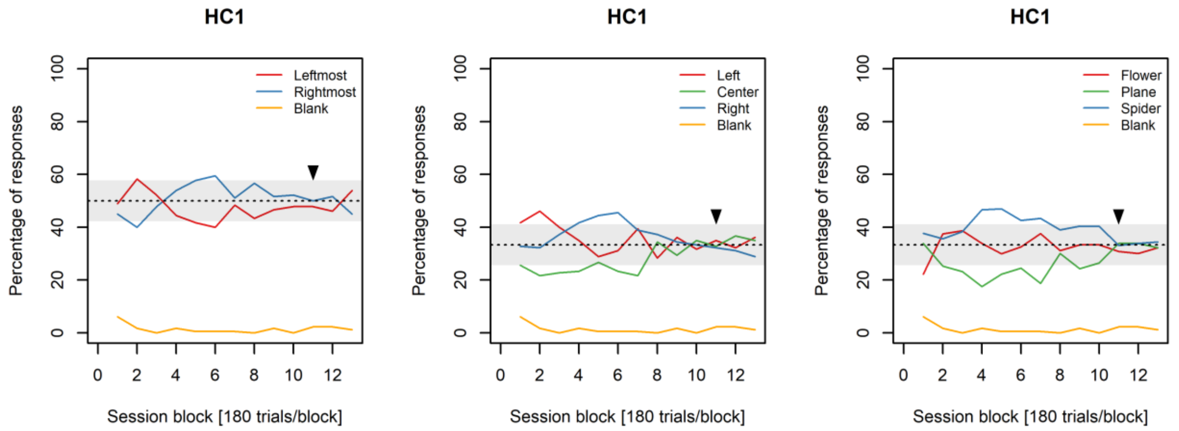

Fig. S7. **Response profiles of young female individual HC_1 during home cage-based training to C_2.** Percentages are presented for session blocks of 5 sessions (180 trials). Dashed black lines represent the expected response probabilities for the presented instances, shaded grey areas include all responses for which the probability is >5% (as based on two-tailed binomial tests: n = 180, alpha = 0.05). Session blocks in which this area was left were considered as biased for the represented instances. Black arrowheads indicate the session block in which C_1 would have been reached. **Left:** Individual percentages of responses made to either the leftmost or the rightmost of the two simultaneously presented items (relative position). **Centre:** Individual percentages of responses made to the stimulus presented in either the left RW, the centre RW, or the right RW (absolute RW location). **Right:** Individual percentages of responses made to either the flower, the plane, or the spider (item).

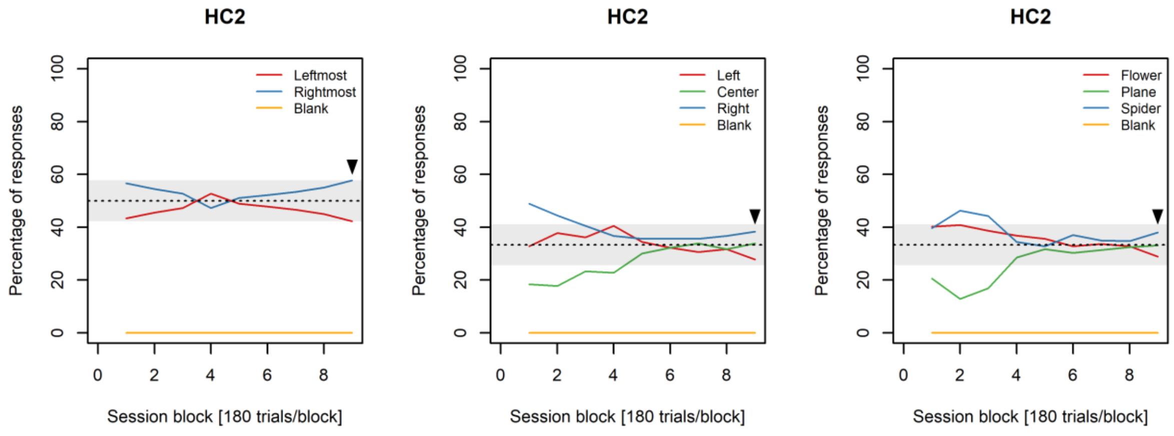

Fig. S8. **Response profiles of young male individual HC_2 during home cage-based training to C_2.** Percentages are presented for session blocks of 5 sessions (180 trials). Dashed black lines represent the expected response probabilities for the presented instances, shaded grey areas include all responses for which the probability is >5% (as based on two-tailed binomial tests: n = 180, alpha = 0.05). Session blocks in which this area was left were considered as biased for the represented instances. Black arrowheads indicate the session block in which C_1 would have been reached. **Left:** Individual percentages of responses made to either the leftmost or the rightmost of the two simultaneously presented items (relative position). **Centre:** Individual percentages of responses made to the stimulus presented in either the left RW, the centre RW, or the right RW (absolute RW location). **Right:** Individual percentages of responses made to either the flower, the plane, or the spider (item).

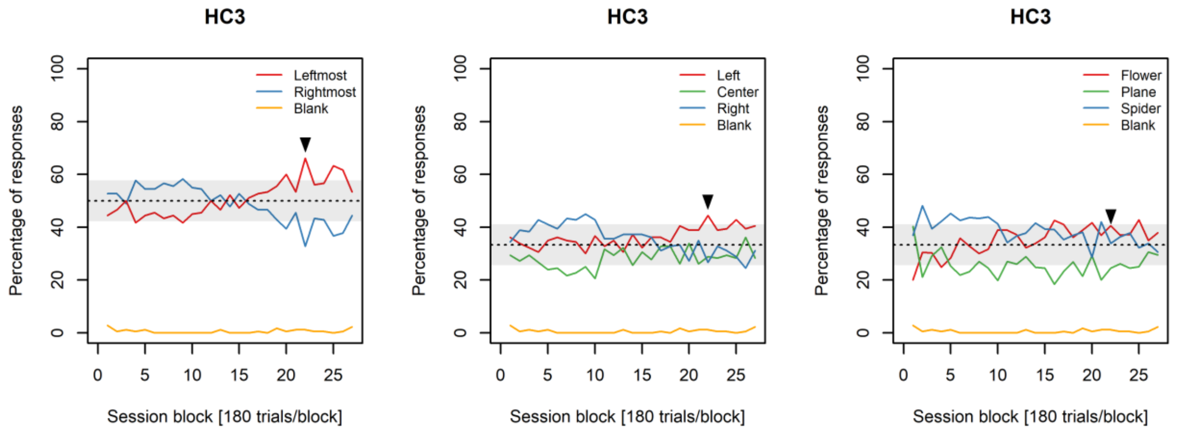

Fig. S9. **Response profiles of young male individual HC_3 during home cage-based training to C_2.** Percentages are presented for session blocks of 5 sessions (180 trials). Dashed black lines represent the expected response probabilities for the presented instances, shaded grey areas include all responses for which the probability is >5% (as based on two-tailed binomial tests: n = 180, alpha = 0.05). Session blocks in which this area was left were considered as biased for the represented instances. Black arrowheads indicate the session block in which C_1 would have been reached. **Left:** Individual percentages of responses made to either the leftmost or the rightmost of the two simultaneously presented items (relative position). **Centre:** Individual percentages of responses made to the stimulus presented in either the left RW, the centre RW, or the right RW (absolute RW location). **Right:** Individual percentages of responses made to either the flower, the plane, or the spider (item).

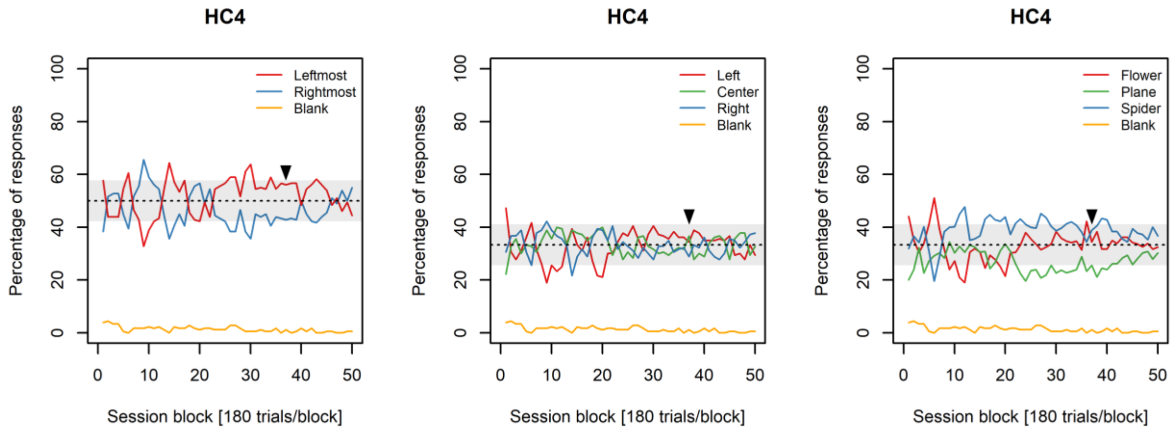

Fig. S10. **Response profiles of old male individual HC_4 during home cage-based training to C_2.** Percentages are presented for session blocks of 5 sessions (180 trials). Dashed black lines represent the expected response probabilities for the presented instances, shaded grey areas include all responses for which the probability is >5% (as based on two-tailed binomial tests: n = 180, alpha = 0.05). Session blocks in which this area was left were considered as biased for the represented instances. Black arrowheads indicate the session block in which C_1 would have been reached. **Left:** Individual percentages of responses made to either the leftmost or the rightmost of the two simultaneously presented items (relative position). **Centre:** Individual percentages of responses made to the stimulus presented in either the left RW, the centre RW, or the right RW (absolute RW location). **Right:** Individual percentages of responses made to either the flower, the plane, or the spider (item).

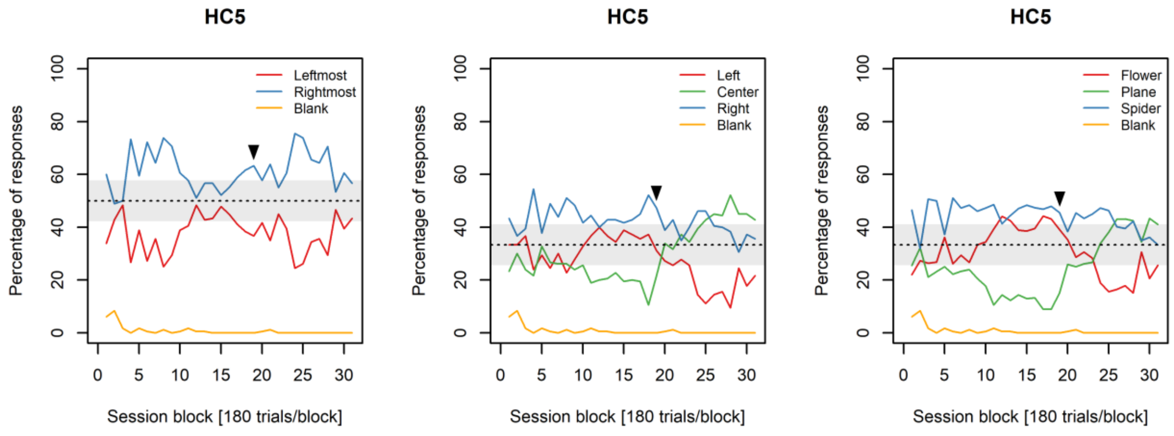

Fig. S11. **Response profiles of old male individual HC_5 during home cage-based training to C_2.** Percentages are presented for session blocks of 5 sessions (180 trials). Dashed black lines represent the expected response probabilities for the presented instances, shaded grey areas include all responses for which the probability is >5% (as based on two-tailed binomial tests: n = 180, alpha = 0.05). Session blocks in which this area was left were considered as biased for the represented instances. Black arrowheads indicate the session block in which C_1 would have been reached. **Left:** Individual percentages of responses made to either the leftmost or the rightmost of the two simultaneously presented items (relative position). **Centre:** Individual percentages of responses made to the stimulus presented in either the left RW, the centre RW, or the right RW (absolute RW location). **Right:** Individual percentages of responses made to either the flower, the plane, or the spider (item).

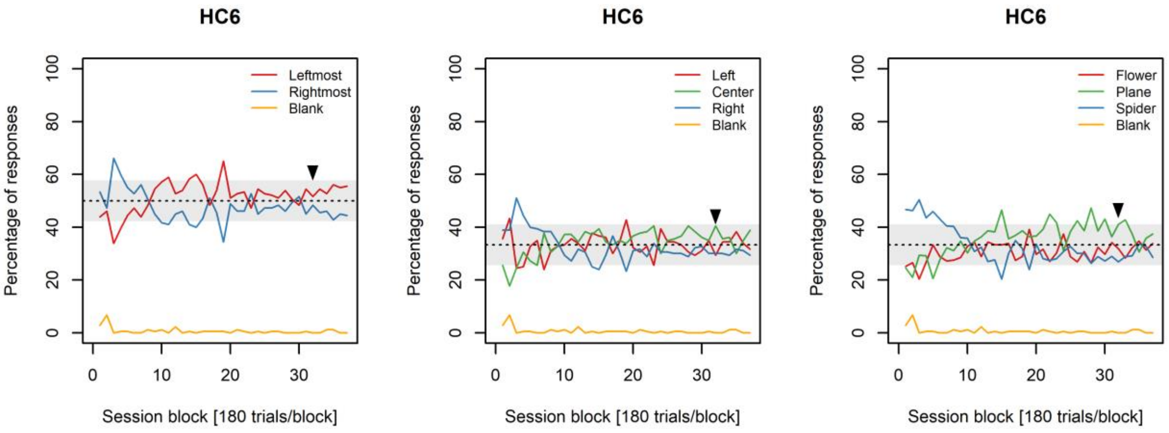

Fig. S12. **Response profiles of old female individual HC_6 during home cage-based training to C_2.** Percentages are presented for session blocks of 5 sessions (180 trials). Dashed black lines represent the expected response probabilities for the presented instances, shaded grey areas include all responses for which the probability is >5% (as based on two-tailed binomial tests: n = 180, alpha = 0.05). Session blocks in which this area was left were considered as biased for the represented instances. Black arrowheads indicate the session block in which C_1 would have been reached. **Left:** Individual percentages of responses made to either the leftmost or the rightmost of the two simultaneously presented items (relative position). **Centre:** Individual percentages of responses made to the stimulus presented in either the left RW, the centre RW, or the right RW (absolute RW location). **Right:** Individual percentages of responses made to either the flower, the plane, or the spider (item).

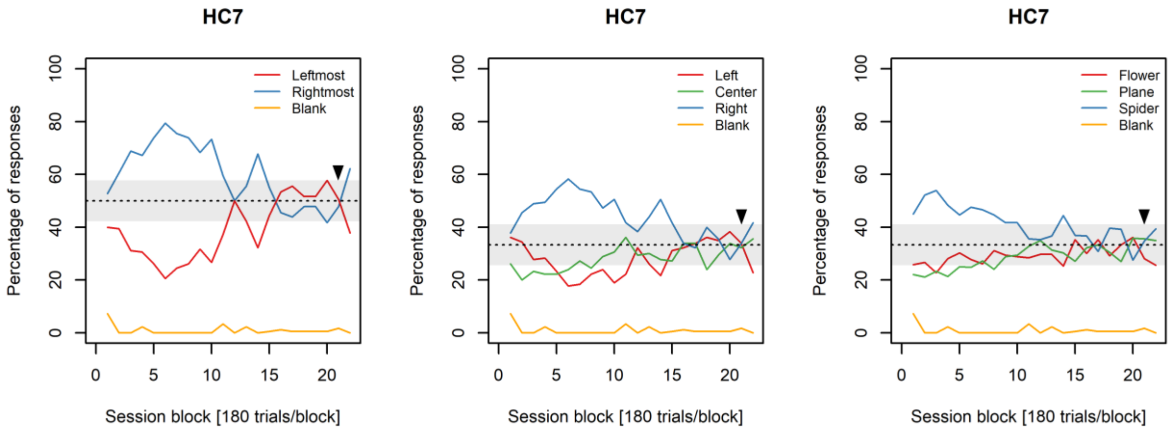

Fig. S13. **Response profiles of old female individual HC_7 during home cage-based training to C_2.** Percentages are presented for session blocks of 5 sessions (180 trials). Dashed black lines represent the expected response probabilities for the presented instances, shaded grey areas include all responses for which the probability is >5% (as based on two-tailed binomial tests: n = 180, alpha = 0.05). Session blocks in which this area was left were considered as biased for the represented instances. Black arrowheads indicate the session block in which C_1 would have been reached. **Left:** Individual percentages of responses made to either the leftmost or the rightmost of the two simultaneously presented items (relative position). **Centre:** Individual percentages of responses made to the stimulus presented in either the left RW, the centre RW, or the right RW (absolute RW location). **Right:** Individual percentages of responses made to either the flower, the plane, or the spider (item).

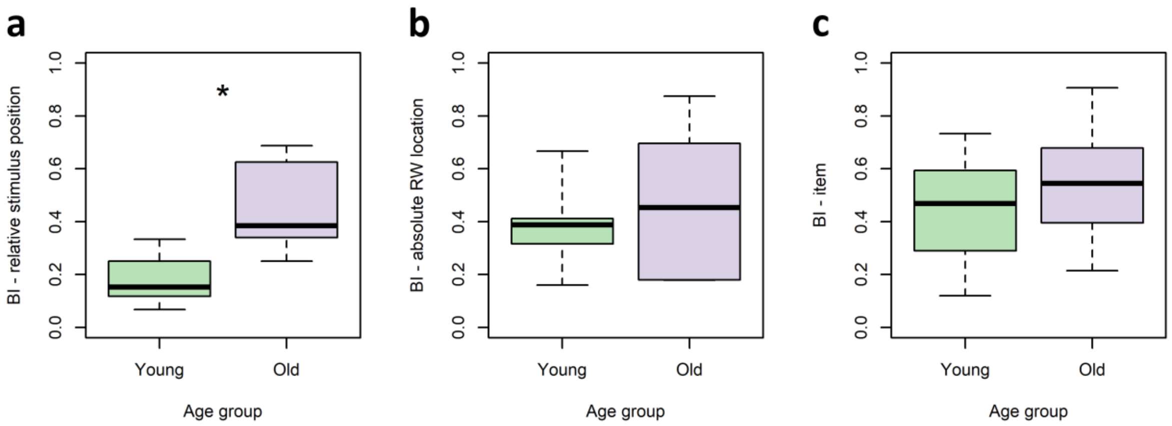

Fig. S14. **Elemental response strategies and age. a** Group-level comparison of individual relative stimulus position (leftmost, rightmost) bias indices. **b** Group-level comparison of individual absolute response window location (left RW, centre RW, right RW) bias indices. **c** Group-level comparison of individual item (flower, plane, spider) bias indices. **a-c** Green = young individuals; purple = old individuals. Horizontal black lines represent the sample median for each boxplot, the belonging boxes represent the inter-quartile-range (IQR). Whiskers extend to the respective sub-sample extremes. Significance code: *$p < 0.016$; Two-tailed Wilcoxon rank sum tests.
