# Supplementary material for: Age affects procedural paired-associates learning in the grey mouse lemur (Microcebus murinus)
Source: Sci Rep. 2021 Jan 13;11:1252. doi: 10.1038/s41598-021-80960-y (PMC7806666; doi:10.1038/s41598-021-80960-y)
Supplement: Supplementary file 2 — Supplementary Information 2. [file 41598_2021_80960_MOESM2_ESM.pdf]

Age affects procedural paired-associates learning in the grey mouse lemur (*Microcebus murinus*)

Daniel Schmidtke[1]

[1]Institute of Zoology, University of Veterinary Medicine Hannover, Hannover, Germany

Supporting table

Tab. S1. Individual error profiles during sPAL challenge sessions (120 trials). For each individual, the table lists the total number of errors made as well as the number of errors made separated by the individual stimulus combination sets with either the flower, the plane, or the spider as rewarded S^+. In addition, the table lists p-values from one-sided binomial tests, indicating whether the observed error frequencies for each stimulus set and individual were significantly increased given an expected chance-level of 33.3% for each stimulus set. Bold p-values indicate significance at an alpha-level of 0.05.

Individual	Total	Flower	p_{Flower}	Plane	p_{Plane}	Spider	p_{Spider}
**HC_1**	28	5	0.977	16	**0.007**	7	0.866
**HC_2**	37	12	0.590	18	**0.034**	7	0.981
**HC_3**	21	3	0.986	13	**0.006**	5	0.872
**HC_4**	30	8	0.823	20	**<0.001**	2	1.000
**HC_5**	23	8	0.505	12	**0.045**	3	0.993
**HC_6**	30	7	0.910	14	0.083	9	0.701
**HC_7**	21	0	1.000	17	**<0.001**	4	0.951
