# Supplementary figures and images for: Age affects procedural paired-associates learning in the grey mouse lemur (Microcebus murinus)
Source: Sci Rep. 2021 Jan 13;11:1252. doi: 10.1038/s41598-021-80960-y (PMC7806666; doi:10.1038/s41598-021-80960-y)

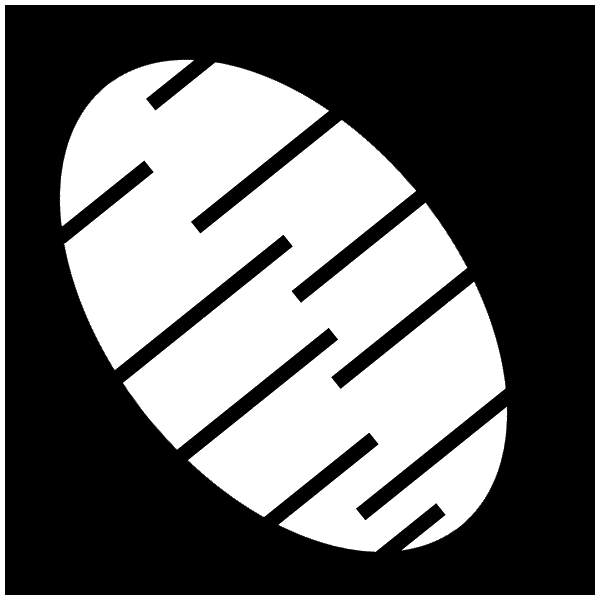

Supplement: Supplementary file 4 — Supplementary Information 4. [file 41598_2021_80960_MOESM4_ESM.bmp]

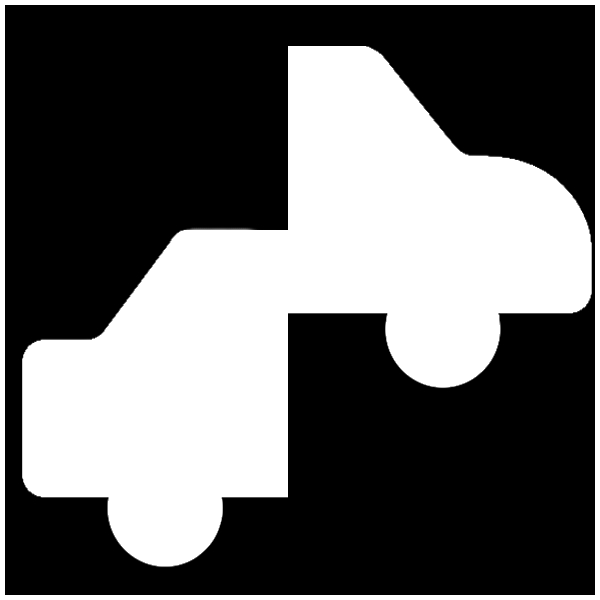

Supplement: Supplementary file 5 — Supplementary Information 5. [file 41598_2021_80960_MOESM5_ESM.bmp]

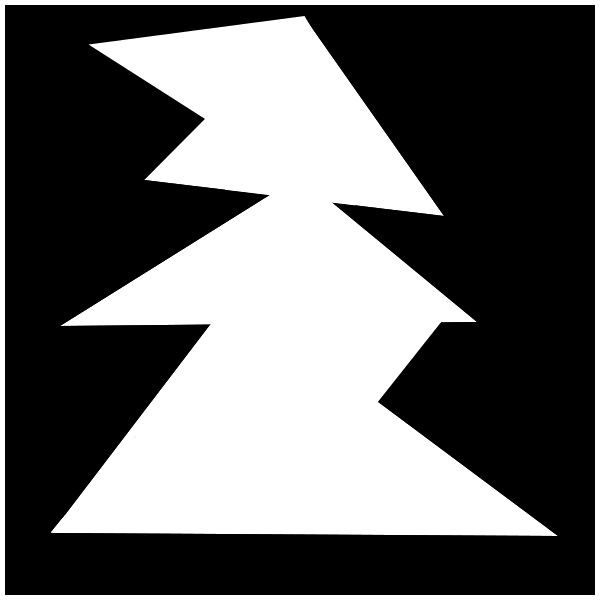

Supplement: Supplementary file 6 — Supplementary Information 6. [file 41598_2021_80960_MOESM6_ESM.bmp]
